# Impact of the Recanalization Level and the First-Pass Effect on Functional Outcomes in Patients After M2 MCA Occlusion Thrombectomy

**DOI:** 10.3390/jcm14082563

**Published:** 2025-04-08

**Authors:** Stefan Pataky, Jakub Fedorko, Piotr Pedowski, Matej Skorvanek, Zuzana Gdovinova

**Affiliations:** 1 Department of Radiodiagnostics and Imaging Techniques, L. Pasteur University Hospital, 04011 Košice, Slovakia; piotr.pedowski@unlp.sk; 2 Department of Radiodiagnostics and Imaging Techniques, Faculty of Medicine, P.J. Safarik University, 04011 Košice, Slovakia; 3 Department of Neurology, Faculty of Medicine, P.J. Safarik University, 04011 Košice, Slovakia; mskorvanek@gmail.com (M.S.); zuzana.gdovinova@upjs.sk (Z.G.); 4 Department of Neurology, L. Pasteur University Hospital, 04011 Košice, Slovakia

**Keywords:** acute ischemic stroke, MCA M2 segment, mechanical thrombectomy, distal and medium vessel occlusion

## Abstract

**Objective**: Acute ischemic stroke (AIS) remains one of the most common causes of death and disability in the world. Mechanical thrombectomy (MT) is the modality of choice in the treatment of AIS and large vessel occlusion (LVO). The endovascular treatment of medium and distal vessel occlusions (DMVO) is currently under intensive scientific investigation. The aim of our study was to prove the feasibility, effectiveness and safety of MT in patients with a primary, isolated occlusion of the M2 segment of the middle cerebral artery (MCA), with a focus on the recanalization level and the first-pass effect (FPE) as predictors. **Methods**: We prospectively assessed patients after MT for primary isolated occlusion of the M2 MCA segment that were treated at our center during a three-year period between July 2021 and June 2024. Our final cohort included 137 patients who met the inclusion criteria. Epidemiological, clinical and technical data, as well as the clinical and safety outcomes of MT procedures, were recorded and analyzed. The primary outcome was defined as a modified Rankin scale (mRS) score of 0–2. Secondary outcomes included excellent functional independence (mRS 0–1) and successful recanalization, defined by a modified thrombolysis in cerebral infarction (mTICI) score of 2c–3. Safety outcomes included symptomatic intracerebral hemorrhage (sICH), any intracerebral (IC) hemorrhage and 90-day mortality. **Results**: The mean age of our cohort was 71.8 ± 12.5 years; 59 were men (43.1%). The primary outcome (mRS 0–2) was achieved in 89 (65%) patients. An excellent functional outcome (mRS 0–1) was reached in 58 (42.3%) and successful recanalization (mTICI 2c–3) in 118 (86.1%) patients. sICH was present in 5 cases (3.7%), any IC hemorrhage in 42 (30.7%) and 90-day mortality in 28 (20.4%). We found a statistically significant correlation between the primary outcome (mRS 0–2) and a successful recanalization mTICI of 2c–3 (*p*—0.024). This correlation was even stronger between excellent functional outcomes and a recanalization mTICI of 2c-3 (*p* < 0.001). The study did not confirm the importance of the first-pass effect (FPE) during MT of the M2 segment (*p*—0.489). We also noticed a significant 31.3% mortality increase in the group of patients in which recanalization of the occluded M2 branch was insufficient. **Conclusions**: MT is a powerful and effective treatment method for AIS caused by an occlusion of the M2 segment in real-life conditions. Patients have a higher probability of a long-term good functional outcome when complete or near-complete reperfusion is achieved.

## 1. Introduction

Numerous randomized controlled trials (RCTs) have proved the efficacy and safety of mechanical thrombectomy (MT) in patients with large vessel occlusions (LVO) in the anterior circulation. The goal of MT is to achieve reperfusion of the affected brain tissue as early as possible to maximize the probability of good functional clinical outcomes [1].

Major RCTs have considered successful reperfusion as a modified thrombolysis in cerebral infarction (mTICI) scale value ≥ 2b, which is defined as ante-grade reperfusion of more than half of the previously occluded target artery territory. However, near-complete reperfusion, a value of mTICI 2c (>90% reperfusion of the downstream territory) and complete reperfusion (mTICI 3) are associated with greater neurological improvements during hospitalization, a better functional outcome at 90 days and reduced infarct growth [2,3,4]. Achieving mTICI 2c or 3 is the aim of MT for anterior circulation^4^. Restoring a complete or near-complete reperfusion (mTICI 2c–3) in a single pass of MT is defined as the first-pass effect (FPE) and has been proven to be an independent predictor of good clinical outcomes in patients with occlusion of the M1 segment of the MCA [5,6,7]. FPE is also associated with a lower use of healthcare resources and lower estimated costs [7].

### Distal and Medium Vessel Occlusion

All the available RCTs until now have focused on LVO. However, 24% to 40% of acute ischemic strokes are caused by medium and distal vessel occlusions (DMVOs) [8].

The clinical benefit of MT for M2 occlusions has been controversial for a long time [9]. Despite less severe clinical symptoms than proximal occlusions, DMVOs, particularly those in the dominant cerebral hemisphere, can cause significant morbidity and disability [10]. Emerging data suggest that MT might also be safe and effective for medium and distal occlusions [11]. Furthermore, MT has recently been emphasized by an international consensus of experts as an encouraging option for medium and distal occlusions and is increasingly performed for medium and distal occlusion AIS worldwide [12]. However, since there are significant anatomical differences between the M1 and M2 MCA segments and big randomized controlled trials are yet to be published, it is not clear whether all the benefits of MTE for LVO in anterior circulation can be extrapolated to patients with M2 occlusion of the MCA. Moreover, predicting the value of FPE on good clinical outcomes in the case of isolated M2 occlusion has not been sufficiently studied.

Successful recanalization has been shown to be an independent predictor of favorable functional outcomes for DMVOs in the MCA territory. Nevertheless, levels of recanalization after MT in patients with DMVOs can vary. Most of the available studies focus on comparing MT results at the level of the M1 and M2 MCA segments and consider an mTICI ≥2b successful [9,13]. However, complete or near-complete reperfusion (mTICI 2c–3) should be the aim of every MT, as these patients are most likely to achieve better functional outcomes compared with patients with TICI ≤ 2b reperfusion. The study of Aoki et al. suggested that complete recanalization, defined as mTICI 3, is associated with favorable outcomes in patients with M2 occlusion, while near-complete recanalization (mTICI 2c) did not show the same correlation [14]. To our knowledge there is no comprehensive study that has analyzed MT of the M2 segment of the MCA with the maximalist reperfusion aim and considering only mTICI 2c–3 recanalization levels successful, with an impact on the long-term functional outcome and safety outcomes, including mortality.

The aim of our study was to determine whether the rate of reperfusion had a significant effect on good clinical outcomes, defined as a modified Rankin scale (mRS) score of 0–2 in real-life conditions. We also evaluated first-pass mTICI 2c–3 as an independent predictor of a good clinical outcome (mRS 0–2) and an excellent clinical outcome (mRS 0–1), similar to the findings for MT of the M1 MCA segment. We tried to determine the safe number of MT attempts that still provide clinical benefits for the patients and to clarify whether increasing the number of MT attempts on the M2 segment significantly affects the risk of hemorrhagic complications.

## 2. Methods

### 2.1. Study Design and Oversight

We conducted a single center, prospective study with real-life patients’ data. The study was approved by the Ethics Committee of Louis Pasteur University Hospital in Košice (2021/EK/05027 and 2023/EK/11061). All procedures were in accordance with the principles of the Declaration of Helsinki. Written informed consent was obtained before treatment from all participants who were able to understand the information and sign the form; otherwise, written consent was expected in line with general clinical standards.

### 2.2. Participants

In our study, we prospectively analyzed real-life data from patients treated in a single, comprehensive, endovascular center located in Kosice, Slovakia. Our center serves as a thrombectomy center for 13 primary stroke hospitals, covering a population of approximately 1.6 million people. Data were gathered over three years, from July 2021 to June 2024.

The inclusion criteria for enrolling patients were defined as follows: 1. diagnosis of an acute ischemic stroke in the anterior circulation caused by isolated M2 MCA occlusion; 2. a National Institutes of Health stroke scale (NIHSS; range of 0 to 42, with higher scores representing more severe neurological deficit) score ≥ 5, or aphasia if the NIHSS score was < 5; 3. no intracerebral hemorrhage; 4. an absence of evident large infarct in the correlating M2 territory; 5. presence of a clinically relevant neurological deficit, and the time window was either 6 h or there was proof of salvageable brain parenchyma based on CT perfusion; 6. functional independence, defined by a modified Rankin scale (mRS) of 0–3 prior to stroke onset [15]; 7. a multidisciplinary decision to treat the patient by MT, with or without the prior application of intravenous thrombolysis; 8. availability of baseline patient characteristics and clinical assessments at baseline and at 90 days.

### 2.3. Interventional Procedures

The initial indication was determined by multidisciplinary team consensus among the interventional neuroradiologist, an onsite neurologist and the anesthesiologist in our center. Treatment was performed in routine clinical settings by three interventional neuroradiologists in a single comprehensive stroke center. The strategy of the endovascular procedure was based on the best clinical knowledge of the operators. The aim of the treatment was to achieve the best possible reperfusion with the fewest possible passes of MT while keeping the procedure as safe as possible.

The endovascular treatment (ET) of anterior circulation AIS was performed in routine practice with a maximalist approach to achieve the best possible first-pass effect. Cannulation of the right femoral artery was performed using a modified Seldinger method, in which a 5F arterial sheath was inserted. Then, an 80 cm 6F long sheath was placed in the appropriate position of the common carotid artery, followed by digital subtraction angiography (DSA) at an anteroposterior, lateral and ipsilateral 45° oblique, if necessary, to find the best operating position. In the majority of cases, biplane imaging was used.

**Figure 1:**
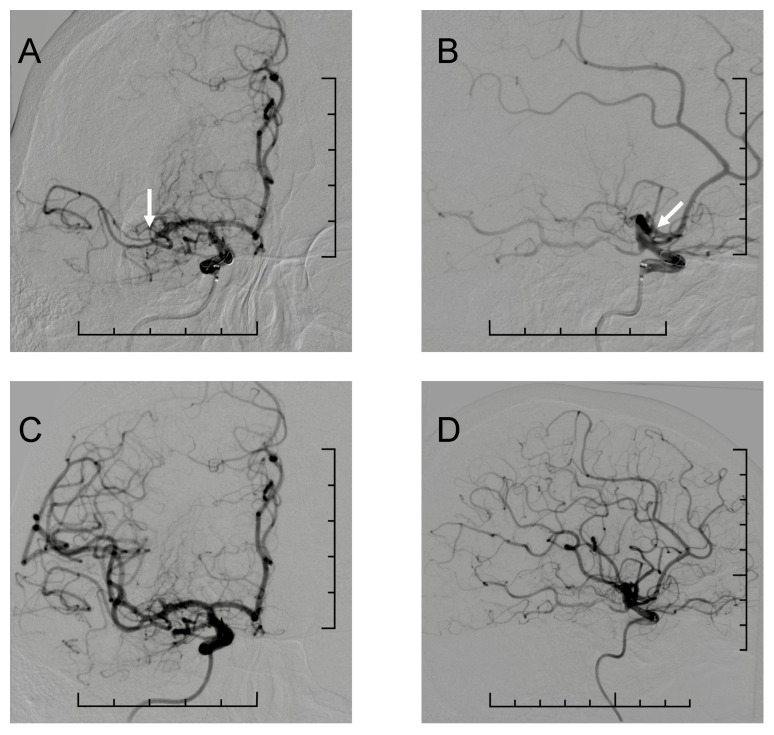
Illustrative case of mechanical thrombectomy for a patient with stroke with isolated M2 segment occlusion of the right middle cerebral artery. (**A**,**B**) The initial cerebral angiography shows the isolated M2 segment occlusion ((**A**)—anteroposterior and (**B**)—lateral views). The white arrow indicates the occlusion of the superior M2 branch of the right MCA. (**C**,**D**) Complete recanalization of the occluded M2 segment (mTICI 3) reached after MT ((**C**)—anteroposterior and (**D**)—lateral view). Scale bars are included in each panel for reference (visible along the right and bottom margins); each scale represents 1 cm.

Stent thrombectomy: The tip of a 6F or 5F aspiration catheter was placed at the C3–4 segment of the internal carotid artery (ICA) under the support of a 0.014-inch microguidewire and a 0.021-inch or 0.017-inch microcatheter. After crossing the occluded segment of the MCA with the microguidewire, the microcatheter tip was introduced at the distal end of the M2 segment; subsequently, the microguidewire was withdrawn and a stent retriever was released in the correct position. The aspiration catheter was moved as close as possible to the proximal end of the occluded segment, and aspiration was initiated. The stent, microcatheter and aspiration catheter were simultaneously and slowly withdrawn under negative pressure suction. The DSA was reviewed after MT, and successful recanalization was confirmed in the presence of an mTICI score of 2c–3. If the vessel recanalization failed, MT with the stent was performed again according to the method mentioned above. On the basis of the fact that in the M1 segment, reperfusion after MT at the level of mTICI 2c–3 has been proven to achieve better clinical improvement in 24 h NIHSS scores and has had a significantly better functional outcome, with mRS 0–1 at 90 days [16], this was also defined as successful reperfusion in our analysis. Illustrative case of a patient with isolated M2 segment occlusion of the right MCA and consequent mTICI3 complete reperfusion after mechanical thrombectomy is shown on Figure 1. 

There is no established gold standard for the anesthetic management of patients undergoing MT. There are three possible anesthetic techniques used in daily practice:

1. Local anesthesia.
2. Monitored anesthetic care (conscious sedation administered by an anesthesiology team), which can be beneficial for the patient, especially because of more stable blood pressure levels, the possibility of continuously monitoring neurological status and a shorter time to recanalization. On the other hand, sedative agents may potentially compromise the patient’s airway. As these are emergency cases, there is a risk of aspiration. Also, the involuntary movement of the patient may prolong the procedure.
3. General anesthesia [17].

To provide safe catheterization of the distal segments of the MCA, the majority of cases (82.5%) were performed under general anesthesia. Distal catheterization itself is more challenging than proximal M1 navigation because of the smaller diameter of the vasculature, increased tortuosity and more severe angulations. Precise roadmaps require patients to lie perfectly still, which is often impossible for stroke patients without general anesthesia. Anatomical variability in this area and also possible clot localization at the origin of the M2 branches can be challenging and sometimes require a neurointerventional radiologist to blindly explore various branches of the MCA with the microwire. To minimize the possible harm that can be caused by this maneuver, general anesthesia is useful. Furthermore, a patient’s involuntary movements can result in device dislocation, which can prolong the time needed to reach reperfusion and might also lead to an increased rate of adverse events, such as clot migration, vessel dissection, perforation or intracerebral hemorrhage [18].

### 2.4. Measures

The analyzed data included pre-stroke functional status, patients’ medical history and radiological parameters such as the ASPECTS score [19], collateral score [20] and M2 dominance. We collected detailed procedural data (including the materials and devices used for intervention). Neurological status on admission and at discharge along with estimated pre-stroke functional independence and reperfusion levels were also part of the dataset. Functional independence was assessed and recorded after three months, together with 90-day mortality.

Neurological status was determined using the NIHSS at baseline and at discharge by the neurologist on duty. Functional outcomes were defined by the mRS. Pre-stroke functional independence was estimated by the onsite neurologist. Reperfusion levels and M2 MCA dominance were evaluated post procedurally by the treating interventional radiologist based on the DSA images. To assess reperfusion levels, the mTICI scale was used. M2 dominance was determined based on anatomical features. The three-month post-treatment functional independence status was assessed by a phone call by the study investigators or in person by a local neurologist.

### 2.5. Outcomes

The primary outcome parameter of the study was good functional status (defined as an mRS score ≤ 2 at 90 days); secondary outcomes were defined as successful recanalization (mTICI 2c or mTICI 3) and an excellent functional outcome (mRS 0–1). Safety outcomes included symptomatic intracerebral hemorrhage (sICH), defined using the SITS-MOST definition [21], any intracerebral (IC) hemorrhage and mortality after 90 days (an mRS score of 6).

### 2.6. Statistical Analysis

For statistical analysis, we used SPSS software (version 22.0; IBM, Chicago, IL, USA). Standard descriptive analysis using percentages, means and medians was used to describe raw outcome data and all endpoints. Statistical tests for the determination of significant differences between compared groups of variables included Pearson’s Chi-squared test, the *t*-test, the Mann–Whittney U test and univariate binary logistic regression. To address the normality of distribution among continuous variables, we used visual methods (mainly histograms with a normal distribution curve), and if any discrepancy occurred, we performed the Shapiro–Wilk test. For more precise statistical analysis, some ordinal and scale variables had to be dichotomized. The primary outcome was made binary as mRS 0–2 versus mRS 3–6. Secondary outcomes were determined by successful vessel recanalization, defined by an mTICI score of 2c or 3 versus a group of patients with an mTICI score of 2b or less; the excellent clinical outcome group consisted of patients with mRS 0–1. Safety outcomes were defined by the binary categorical variable of any IC hemorrhage, sICH and 90-day mortality (present or absent).

## 3. Results

We identified 209 patients that were treated for M2 MCA occlusion over the study period. In order to achieve a homogeneous group of patients for analysis, we excluded 21 patients who presented with tandem occlusions (ostial ICA and M2 MCA). We also excluded 45 patients with distal embolization who initially presented with intracranial ICA or M1 MCA occlusion. This group of patients included M2 occlusions after i.v. thrombolysis or distal embolization during mechanical thrombectomy. Finally, we excluded six patients who presented with combined occlusions (M2 MCA + anterior cerebral artery). After data selection, our patient group consisted of 137 individuals who were treated in our center with primary isolated M2 MCA occlusion.

**Figure 2:**
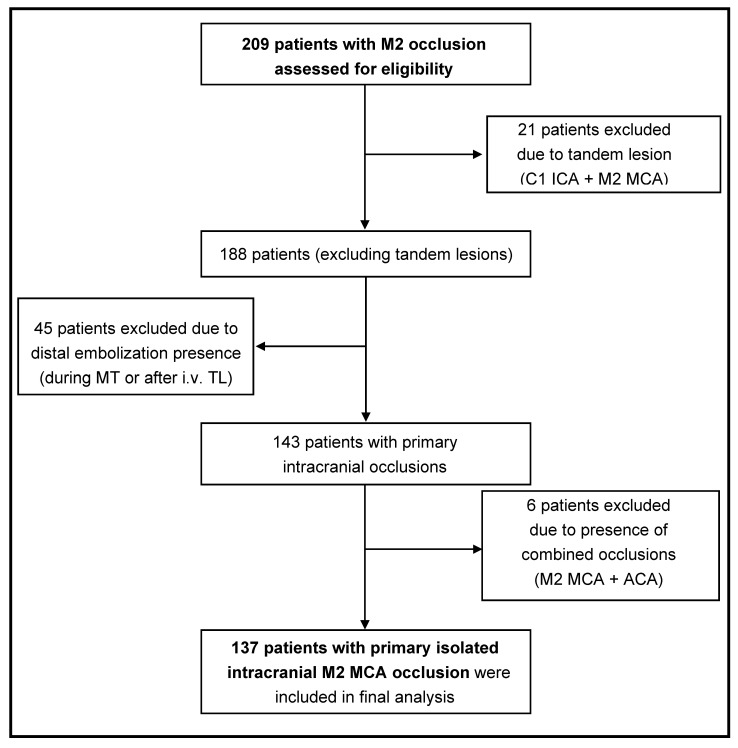
Screening and enrollment process and patient group identification.

The screening and enrollment process is summarized in Figure 2.

### 3.1. Basic Epidemiological Parameters

The mean age in our cohort was 71.8 ± 12.5 years. The mean admission NIHSS was 11.6 ± 5.3, with a median of 11. The rate of i.v. thrombolysis was 38%. The reason for not administering i.v. thrombolysis was predominantly due to an advanced time window or concomitant anticoagulation therapy. Other basic epidemiological parameters (divided into two groups based on FPE) are summarized in Table 1.

### 3.2. Primary and Secondary Outcomes

**Figure 3:**
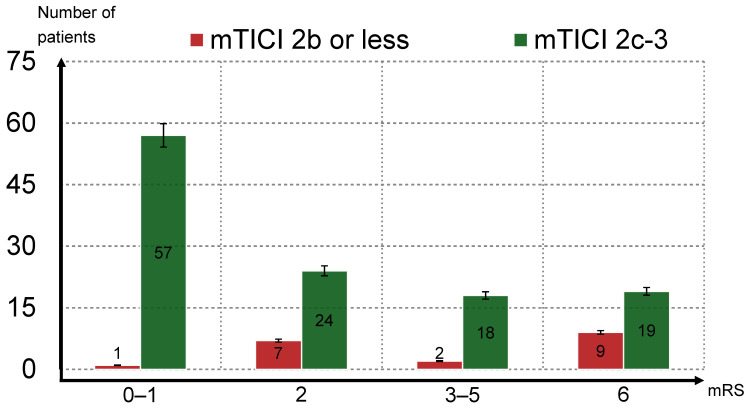
mRS outcome distribution divided into subgroups based on the level of reperfusion. Legend: The x axis illustrates four groups of patients divided based on mRS; the y axis represents the number of patients. Groups of patients who reached reperfusion levels of mTICI 2b or less are depicted in red; patients who reached reperfusion levels of mTICI 2c or mTICI 3 are depicted in green.

We achieved a good reperfusion level (mTICI 2c–3) in 118 cases (86.1%). From this group, 81 (68.6%) patients had a good clinical outcome (mRS 0–2), 57 (48.3%) patients had an excellent clinical outcome and 37 (31.4%) individuals had mRS 3–6 at 90 days. In the group of 19 patients where good reperfusion was not achieved (mTICI 2b or less), 8 (42.1%) had a good clinical outcome, 1 (5.3%) had an excellent clinical outcome and 11 (58%) patients had a bad clinical outcome. The correlation between a good clinical outcome and a good reperfusion level was statistically significant (*p* = 0.024) and was even stronger between an excellent clinical outcome and a good reperfusion level (mTICI 2c–3) (*p* < 0.001). General characteristics of outcome divided by mTICI grade are sumarized in Table 2, distribution of outcome based on level of reperfusion is depicted in Figure 3.

In our group of 137 patients with M2 MCA occlusion, we were able to achieve mTICI 2c–3 recanalization levels after the first pass (FP) of MT in 63 (46%) cases (FPE group). In 74 individuals (54%), successful recanalization was not achieved after the first pass, and multiple passes of thrombectomy were performed.

In the FPE group, 39 (61.9%) patients had a good clinical outcome at the 3-month follow-up (mRS 0–2), 31 (49.2%) patients had excellent clinical outcomes and 24 (38.1%) individuals had mRS 3–6. In the multiple-passes (MP) group, 50 (67.6%) patients had a good clinical outcome, 27 (36.5%) had an excellent clinical outcome and 24 (32.4%) had a bad clinical outcome at 90 days. No statistical significance regarding good clinical outcomes (mRS 0–2) between the FPE and MP of MT groups was shown (*p* = 0.489). Correlation between FPE and excellent clinical outcomes did not reach statistical significance either (*p* value = 0.133). General characteristics of the outcomes divided according to the presence of FPE are sumarized in Table 3.

A total of 101 patients had one or two passes of MT, from which 70 (69.3%) had a good clinical outcome (mRS 0–2) and 31 (30.7%) had mRS 3–6 at 90 days. Of the 70 patients with a good clinical outcome, 51 (50.5%) achieved an excellent mRS (0–1). Three or more passes of MT were performed in 31 patients, 17 (54.8) of whom had a good clinical outcome. Of these, seven had an excellent clinical outcome (22.6%). Fourteen of the 31 patients who underwent multiple-pass MT (45.2%) had a bad clinical outcome, with mRS 3–6 at 90 days. The comparison of groups with 1–2 passes of MT and ≥3 passes of MT in terms of good clinical outcomes did not reach statistical significance (*p* = 0.137), but we found a significantly higher probability of an excellent clinical outcome when we performed one or two MT passes (*p* = 0.006).

### 3.3. Safety Outcomes

An evaluation of 90-day mortality showed that in the group with a good reperfusion level (mTICI 2c–3), mortality was 16.1% (19/118 patients), while in the group with a reperfusion level of mTICI 2b or less, mortality reached 47.4% (9/19 patients). The difference in mortality between the two groups was 31.3%, and the correlation between mortality at 90 days and insufficient reperfusion (mTICI 2b or less) reached statistical significance (*p* = 0.002). In the group of patients where ≥4 thrombectomy passes were performed, we observed ICH in 40% of cases, while the patient group with three thrombectomy passes or fewer had a 30.7% incidence of ICH. Taking into account the size and asymmetry of the thrombectomy groups on the basis of the number of passes and the small sample size in the group with ≥4 thrombectomy passes, the correlations did not reach statistical significance (*p* = 0.47). In the same group of patients, the difference between incidence of sICH and 90-day mortality was not statistically significant.

## 4. Discussion

We examined and retrospectively analyzed the feasibility and safety of MT in 137 patients with an isolated M2 MCA occlusion in our center over a three-year period between July 2021 and June 2024.

By interpreting our results, we were able to come to some interesting conclusions that may further contribute to understanding the complex issue of acute M2 occlusions and the consecutive process of reperfusion. Firstly, to verify and support the efficacy of MT for patients with M2 MCA occlusion, we evaluated the correlation between a good clinical outcome (mRS 0–2), an excellent clinical outcome (mRS 0–1) at 3 months and a good reperfusion level after MT (mTICI 2c–3). We found that successful reperfusion of the occluded territory is significantly linked not only to a better chance of achieving a good functional outcome but also to the chance of an excellent clinical outcome and a sufficient level of independence for the patient. These findings support other results reported in different MT studies that found mTICI 2b–3 recanalization to be an independent parameter of a favorable clinical outcome [13,22,23,24]. Kniep et al. analyzed patients from the German Stroke Registry, which included patients after MT in the M2 segment, and suggested that successful recanalization with mTICI ≥ 2b versus mTICI < 2b increases the probability of a good long-term functional outcome from 27% to 47%, with a number-needed-to-treat (NNT) of 5. For M1 occlusions, the probability of a good outcome increased from 16% to 38%, with an NNT of 4.5. Successful recanalization by MT not only increases the probability of a good functional outcome but even improves the chance of an excellent long-term functional outcome in patients with M2 occlusions [13]. According to Aoki et al., complete recanalization, but not mTICI 2b, was associated with favorable outcomes [14].

Although mTICI 2b–3 has been routinely considered as successful recanalization after EVT, a recent meta-analysis evaluated the association between recanalization degree and clinical outcomes and emphasized the importance of mTICI 3 in order to rescue more patients with a higher probability of mTICI 2b [25,26,27]. Also, in our study, the evaluation of successful reperfusion was considered more strictly, with maximum effort made to reach mTICI 2c–3. In light of technical advances achieved in recent years, providing safer MT of more distal brain regions and significant experience growth with MT procedures, reaching complete or near-complete reperfusion at the level of mTICI 2c–3 should be considered the primary goal of MT.

A second important finding in our dataset was that in our real-life single center cohort, the first-pass effect was not linked to a higher chance of achieving a good clinical outcome (mRS 0–2), although the tendencies suggest that it increases the chance of excellent functional outcomes (mRS 0–1) by 12.7%. The sample size of our cohort was probably the limitation to achieving statistical significance. However, the tendency to achieve an excellent clinical outcome after successful first-pass reperfusion and the decrease in its probability after multiple passes of MT is evident, in our opinion. This implies that further investigation and more multicentric data in this area are required.

FPE was proven to be an independent predictor of good clinical outcomes in patients with M1 MCA occlusion. In contrast with MT in the M1 segment, there is very limited data on FPE in patients with isolated M2 occlusion. An analysis of the multicentric, international database from the STAR collaboration has found that first-pass complete reperfusion persisted as an independent predictor for a favorable outcome only in M1 occlusions, while it was not significant in the subgroup of patients with ICA and M2 occlusions. However, only patients with an mTICI 3 reperfusion rate were selected for the analysis [28]. Finding the specific reason for the fact that FPE is not as important in distal (M2) occlusion as in M1 is, in the current state of knowledge, speculative. Some authors suggest that, in the case of isolated M2 occlusion, a smaller size of downstream territory is affected and, due to shorter routes over the convexity, collaterals might be more sufficient to compensate long term for persisting vessel occlusions; therefore, these patients are still able to achieve good clinical outcomes after multiple passes of MT [13].

Assuming that good reperfusion is crucial for a patient’s prognosis but knowing that, in 54% of cases, multiple passes of thrombectomy were needed to achieve a good reperfusion level, we tried to find the maximum number of thrombectomy attempts after which thrombectomy does not add any additional benefit to the patient and increases the risk of ICH. In our model, we were unable to prove our hypothesis that one or two passes of MT in patients with M2 occlusion significantly increases the chance of achieving a good clinical outcome, though it is likely that the probability of good functional outcomes after two MT passes decreases. However, our study shows that the chance of an excellent functional outcome after one or two passes of MT is significantly higher than in patients with ≥3 passes of MT (*p* value = 0.006). A successful recanalization level of mTICI 2c-3 is often achieved after the first or second pass, while more complex cases require multiple passes and still may not achieve successful recanalization. The reason for this may be significant tortuosity of the vasculature or fragility of the clot, especially after administration of thrombolysis or dissection of the occluded artery.

An analysis of patient outcomes at 90 days in our cohort showed a significant 31.3% increase in mortality in the group where MT was considered to be unsuccessful (mTICI ≤ 2b) and did not provide sufficient levels of reperfusion. This finding is in line with the ETIS registry results, where authors report that the successful reperfusion group showed a lower mortality rate than the unsuccessful reperfusion group (15.4% vs. 38.9%) [29]. However, the authors of the German Stroke Registry did not specifically highlight the mortality differences between the group with good reperfusion (mTICI ≤ 2b) and the group with futile reperfusion (mTICI 2b–3), but from the mRS shift pre-stroke status to 90 days, it is obvious that mortality was at least 20% higher in the group with unsuccessful recanalization [13].

Landmark RCTs [30,31] have confirmed that MT is associated with lower mortality in patients with AIS due to LVO. Our analysis of real-life data suggests that unsuccessful MT in patients with isolated M2 MCA occlusion may lead to a significant increase in mortality. Therefore, successful reperfusion of the M2 segment may have life-saving potential. Higher mortality increases in the case of insufficient reperfusion could potentially be mitigated by significant accessibility increases in long-term social and nursing care and specialized physical and rehabilitation services. Intensive physical therapy with a special focus on the most severely disabled patients can be crucial in lowering the mortality rates [32]. To confirm this hypothesis, more research in this field is necessary.

When performing endovascular procedures in patients with a serious medical condition, safety is critically important. Our findings show that the safety of MT at the M2 level is favorable, with a low incidence of sICH (3.7%). From our data, we assume that a safe number of passes that does not increase the risk of bleeding and 90-day mortality is most probably in the range of three to four passes. Nevertheless, the small sample size of multiple-pass thrombectomies limits the statistical significance of this assumption. This smaller number of cases with ≥ 4 MT passes is probably also due to the reluctance of operators in our center to perform more than four passes in distal thrombectomy cases.

## 5. Strengths and Limitations

The main strength of our study is that the analysis was based on real-life data. This uniquely highlights mTICI 2c–3 as a successful reperfusion level after MT of isolated M2 MCA occlusion and also includes the mortality data as part of a complex analysis.

The main limitation of our study is the monocentric nature of the patient cohort. All the patients were racially uniform (white Caucasian) and came from eastern Slovakia, which limits the generalizability of our results. The sample size turned out to be borderline or small for some subgroup analyses to show statistical significance.

The absence of core lab adjudication of the modified treatment in cerebral ischemia (mTICI) score to quantify the degree of reperfusion after endovascular treatment can also be considered a limitation. The reperfusion level was assessed by local operators after the procedure, and it has been shown that mTICI scores in patients with M2 or M3 occlusion are more likely to be overestimated by operators [33].

## 6. Conclusions

Achieving near-complete or complete reperfusion (mTICI 2c–3) in the endovascular treatment of isolated M2 MCA occlusions appears to be crucial for improvements in functional outcomes and a reduction in mortality. Even if it takes multiple attempts, the final reperfusion grade (rather than the number of passes) most strongly influences survival and functional independence (mRS 0–2). While multiple passes are more likely to result in good outcomes (mRS 0–2), the probability of achieving an excellent outcome (mRS 0–1) declines after about two MT passes. We observed a statistically significant drop-off in mRS 0–1 when patients required ≥3 passes. We found a numerical increase in hemorrhagic events if four or more passes were performed. According to our findings and clinical experience, we suggest cautious performance of MT in cases of isolated M2 MCA occlusion, especially in small M2 vessels, and to consider stopping the procedure if the MCA branch is too tortuous or fragile, the patient is already at high risk of hemorrhage or only marginal improvement in reperfusion is observed after three passes of MT. For the objectivization of decision-making, whether to stop MT or continue, developing a risk calculator based on interventional and anatomical parameters could be very useful. For this development, a larger amount of multicentric data is necessary.

## Figures and Tables

**Table 1:** Basic epidemiological parameters and clinical characteristics.

Parameter	First-Pass mTICI 2c–3 (*n* = 63)	Non-First-Pass mTICI 2c–3 (*n* = 74)	*p* Value
%	46	54	
Males (%)	29 (46)	30 (40.5)	0.764
Females (%)	34 (54)	34 (59.1)	
Age (mean ± SD)	70.6 ± 12.5	72.6 ± 11.1	0.467
Admission NIHSS (median)	11.5	10.0	
Arterial hypertension (%)	51 (81)	65 (87.8)	0.269
Atrial fibrillation (%)—including primo-manifestation	32 (50.8)	38 (51.4)	0.567
Diabetes mellitus (%)	13 (20.6)	18 (24.3)	0.772
Wake-up stroke (%)	8 (12.7)	13 (17.6)	0.430
i.v. Thrombolysis (%)	28 (44.4)	24 (32.4)	0.149
ASPECTS score (mean ± SD/median)	9.4 ± 1.0/10	9.4 ± 1.0/10	
Collateral score (mean ± SD/median)	2.2 ± 0.5/2	2.2 ± 0.6/2	
M2 dominant branch (%)	26 (41.3)	37 (50)	0.307

NIHSS—National Institutes of Health Stroke Scale, i.v.—intravenous, ASPECTS—Alberta Stroke Program Early CT Score.

**Table 2:** General characteristics of outcomes divided by mTICI grade.

Parameter	mTICI 2b or Less (*n* = 19)	mTICI 2c–3 (*n* = 118)	*p* Value
mRS 0–2 (%)	8 (42.1)	81 (68.6)	0.024
mRS 0–1 (%)	1 (5.3)	57 (48.3)	<0.001
Mortality = mRS 6 (%)	9 (47.4)	19 (16.1)	0.002
Any ICH (%)	6 (31.6)	36 (30.5)	0.925
Symptomatic ICH (%)	1 (5.3)	4 (3.4)	0.686

mTICI—modified thrombolysis in cerebral infarction, mRS—modified Rankin scale, ICH—intracerebral hemorrhage.

**Table 3:** General characteristics of the outcomes divided according to the presence of FPE.

Parameter	First-Pass mTICI 2c–3 (*n* = 63)	Non-First-Pass mTICI 2c–3 (*n* = 74)	*p* Value
mRS 0–2 (%)	39 (61.9)	50 (67.6)	0.489
mRS 0–1 (%)	31 (49.2)	27 (36.5)	0.133
Mortality = mRS 6 (%)	12 (19.1)	16 (21.6)	0.710
Any ICH (%)	17 (27)	25 (33.8)	0.390
Symptomatic ICH (%)	3 (4.8)	2 (2.7)	0.522

mTICI—modified thrombolysis in cerebral infarction, mRS—modified Rankin scale, ICH—intracerebral hemorrhage.

## Data Availability

The datasets and images used and/or analyzed during the current study are available from the corresponding authors upon reasonable request.
